# A phase I, single-center, open-label study of RM-1929 photoimmunotherapy in Japanese patients with recurrent head and neck squamous cell carcinoma

**DOI:** 10.1007/s10147-021-01960-6

**Published:** 2021-06-24

**Authors:** Makoto Tahara, Susumu Okano, Tomohiro Enokida, Yuri Ueda, Takao Fujisawa, Takeshi Shinozaki, Toshifumi Tomioka, Wataru Okano, Merrill A. Biel, Kosuke Ishida, Ryuichi Hayashi

**Affiliations:** 1 Department of Head and Neck Medical Oncology, National Cancer Center Hospital East, 6-5-1 Kashiwanoha, Kashiwa, Chiba 277-8577 Japan; 2 Department of Head and Neck Surgery, National Cancer Center Hospital East, Kashiwa, Chiba Japan; 3 Clinical Development, Rakuten Medical Inc., 900 Concar Drive, San Mateo, CA 94402 USA; 4 Clinical Development, Rakuten Medical Japan, K.K., Futako Tamagawa Rise Office, 2-21-1, Tamagawa, Setagaya-ku, Tokyo Japan

**Keywords:** RM-1929 (cetuximab sarotalocan), Photoimmunotherapy, Recurrent head and neck squamous cell carcinoma, Tumor-targeted monoclonal antibodies, Light-activatable dye (IRDye®700DX), Cetuximab-IR700DX conjugate

## Abstract

**Background:**

To determine the safety, preliminary efficacy, pharmacokinetics, and immunogenicity of a single cycle of RM-1929 photoimmunotherapy, an anti-EGFR antibody cetuximab conjugated with a light-activatable dye (IRDye^®^700DX), in Japanese patients with recurrent head and neck squamous cell carcinoma (rHNSCC).

**Methods:**

Patients received a single fixed dose (640 mg/m^2^) of RM-1929 and a fixed light treatment dose (50 J/cm^2^ for superficial illumination; 100 J/cm fiber diffuser length for interstitial illumination). Safety, tumor response (modified RECIST v1.1 by central radiology review), pharmacokinetics, and immunogenicity were evaluated.

**Results:**

Three Japanese patients were enrolled who had failed ≥ 3 prior lines of therapy including radiation, chemotherapy, cetuximab, and immunotherapy. Target lesions were: submental lesion; right superficial cervical node lesion and oropharynx lesion; and external auditory canal lesion. All patients experienced ≥ 1 treatment-emergent adverse event (TEAE), but none were considered dose-limiting. TEAEs were mild to moderate in severity except for one grade 3 application-site pain, which was transient, resolved without sequelae within 24 h, and did not affect study treatment administration. Thirteen of 17 TEAEs reported were possibly or probably related to study treatment. Three patient reports of application-site pain and localized edema were deemed probably related to study treatment. Objective response was observed in two patients (both partial responses). The third patient had disease progression. RM-1929 concentrations and pharmacokinetic parameters were similar in all patients. No patients tested positive for anti-drug antibodies.

**Conclusions:**

RM-1929 photoimmunotherapy showed a manageable safety profile in rHNSCC. Tumor response in these heavily pre-treated patients was clinically meaningful and warrants further investigation.

**Clinical trial registration:**

The trial was registered with the Japanese registry of clinical trials as jRCT2031200133.

**Supplementary Information:**

The online version contains supplementary material available at 10.1007/s10147-021-01960-6.

## Introduction

Head and neck cancers, including cancers of the oral cavity, oropharynx, nasopharynx, hypopharynx, and larynx, are the seventh most common cancer worldwide, accounting for nearly 900,000 new cancer cases each year [1, 2]. In Asia, the number of new cases is estimated at > 550,000 annually, more than half the worldwide total [2], and each year an estimated 300,000 deaths are attributed to the disease in Asia (5.6% of total cancer deaths) [3]. In Japan, the annual incidence and mortality of cancers of the oral cavity, pharynx, and larynx were projected at 27,700 and 8,900, respectively [4]. Head and neck squamous cell carcinoma (HNSCC) accounts for 90% of head and neck cancers [5]. Approximately, 70% of patients with primary HNSCC present with locally or regionally advanced disease (stage III or IV) [6], which recurs in approximately 40–65% of cases after primary therapy with surgery and radiation, with or without chemotherapy [6–10]. Patients with locally advanced HNSCC have a very poor prognosis, with a 5 year survival rate of only 10–50%, depending on the stage and location of the lesion [6]. Furthermore, patients with advanced disease have poor health-related quality of life (HRQoL) due to the physical effects of tumors affecting vital functions relating to speech and swallowing, and esthetic effects that can have profound emotional and social impacts [11].

Patients with progressive or recurrent HNSCC (rHNSCC) have limited treatment options [12, 13]. Combination chemotherapy regimens, including targeted therapies, yield objective response rates of 10–36% [14–16]. Although checkpoint inhibitors, such as nivolumab and pembrolizumab, have shown activity in rHNSCC, response rates and overall survival (OS) remain limited [17–21]. Treatment of locoregional disease in patients with advanced HNSCC improves disease-free survival and generally ensures long-term control [22–24]. However, there remains an unmet need for new treatment options to provide improved tumor response and locoregional control in patients with locoregional recurrent disease.

**Fig. 1:**
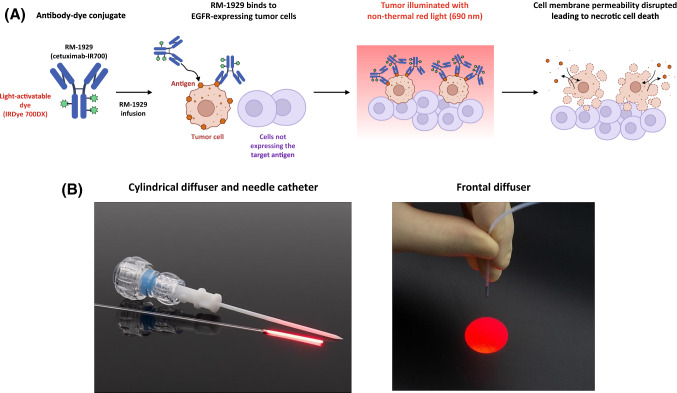
Photoimmunotherapy mechanism of action and RM-1929 photoimmunotherapy overview. **A** Tumor-targeted antibody is conjugated to a light-activatable dye. Following infusion into the body, the tumor is illuminated with non-thermal red light. Preclinical studies demonstrate that light activation leads to anticancer activity mediated by biophysical processes that disrupt the membrane integrity of cells. **B** Tumor illumination is performed approximately 24 ± 4 h after antibody infusion. Cylindrical diffusers placed in needle catheters are used to treat subcutaneous or large tumors, whereas frontal diffusers are used to treat superficial tumors. *EGFR* epidermal growth factor receptor

Photoimmunotherapy utilizes tumor-targeted monoclonal antibodies conjugated with a light-activatable dye (IRDye^®^700DX, abbreviated as IR700) [25]. Preclinical data indicate that activation of the dye with non-thermal red light results in rapid anticancer activity, which is mediated by biophysical processes that disrupt the membrane integrity of tumor cells (Fig. 1A) [26, 27]. In preclinical studies, photoimmunotherapy induced tumor necrosis and immunogenic cell death that can lead to activation of innate and adaptive immunity [28].

RM-1929 (cetuximab sarotalocan) is a first-in-class drug developed on the Illuminox^™^ platform based on photoimmunotherapy [29]. RM-1929 comprises IR700 conjugated with cetuximab, an antibody targeting epidermal growth factor receptor (EGFR), which is well-established to be overexpressed in 80–90% of HNSCC tumors [30, 31]. Moreover, elevated EGFR expression has been associated with a high rate of local recurrence and poor survival expectations [31–33]. RM-1929 photoimmunotherapy requires two steps to be conducted in sequence: (1) intravenous infusion of RM-1929 over 2 h; and (2) tumor illumination with non-thermal red light (690 nm) 24 ± 4 h after infusion. Illumination of the tumor is delivered by frontal diffusers for superficial tumors and by cylindrical diffusers placed in needle catheters inserted into the tumor for large or subcutaneous tumors (Fig. 1B).

In a previous, multicenter, open-label, phase I/IIa, dose-escalation study conducted in the USA in patients with rHNSCC who, in their physician’s opinion, could not be satisfactorily treated with surgery, radiotherapy, or platinum chemotherapy, RM-1929 photoimmunotherapy showed clinically meaningful activity, with a best overall response rate of 43.3% [13/30; 95% confidence interval (CI) 25.5–62.6%], and median OS of 9.30 months (95% CI 5.16–16.92 months) [34]. Treatment was also generally well tolerated. Here we report the findings from a single-center, open-label, phase I study of the safety, preliminary efficacy, pharmacokinetics (PK), and immunogenicity of a single cycle of RM-1929 photoimmunotherapy in Japanese patients with rHNSCC.

## Patients and methods

### Study oversight

The study was conducted in compliance with the protocol, good clinical practice (GCP), guidelines of the International conference on harmonisation (ICH) of technical requirements for registration of pharmaceuticals for human use, and the world medical association Declaration of Helsinki and its most recent amendments. All patients gave written informed consent. The trial is registered at the Japanese registry of clinical trials, identifier: jRCT2031200133.

### Study design and objectives

This was a single-center, open-label, phase I study, which utilized a 3 + 3 design to treat up to six patients. The primary objective was to evaluate the safety of a single treatment cycle of RM-1929 photoimmunotherapy in Japanese patients with rHNSCC who, in their physician’s opinion, could not be satisfactorily treated with surgery, radiotherapy, or platinum-based chemotherapy, and who had no other options for standard of care treatment.

Safety was evaluated via monitoring of dose-limiting toxicities (DLTs), defined as adverse events (AEs) considered related to study treatment as follows: any grade ≥ 3 systemic toxicity other than a hematologic toxicity; grade 4 hematologic toxicity; grade 3 anemia, thrombocytopenia, or neutropenia lasting > 1 week; anemia or thrombocytopenia requiring transfusion; neutropenia requiring hematopoietic factors; alanine (or aspartate transaminase levels > 3 × the upper limit of normal (ULN) and concomitant elevation of bilirubin > 2 × ULN; grade ≥ 3 exposed non-tumor normal soft tissue toxicity that was related to the application of light after administration of RM-1929 photoimmunotherapy.

Key secondary objectives included evaluation of tumor response by modified response evaluation criteria in solid tumors (mRECIST; version 1.1), PK, and immunogenicity.

### Patients

Key inclusion criteria were: histologically confirmed rHNSCC which, in the treating physician’s opinion, could not be satisfactorily treated with surgery, radiotherapy, or platinum chemotherapy and had no other options for standard of care treatment; prior systemic platinum-based chemotherapy for rHNSCC, unless contraindicated or not recommended; life expectancy > 4 months; male or female aged ≥ 18 years; Eastern cooperative oncology group (ECOG) performance status (PS) of 0–2.

Key exclusion criteria were: history of significant cetuximab infusion reactions (grade ≥ 3); tumor invading a major blood vessel (e.g. carotid artery) unless embolized, stented, or surgically ligated to prevent hemorrhage; location and extension of tumor precluded effective photoimmunotherapy; impaired hepatic function [alkaline phosphatase (hepatic), alanine or aspartate transaminase levels > 3 × ULN, and total serum bilirubin > 2 mg/dL]; impaired renal function (serum creatinine > 2 mg/dL).

Further details are provided in Online Resource 1.

### Treatment

A challenge dose of cetuximab 100 mg was administered over 30 min to assess patient tolerability to RM-1929 photoimmunotherapy (cetuximab-IR700 conjugate). Pre-treatment with intravenous dexamethasone and d-chlorpheniramine was used to limit the risk of hypersensitivity.

Patients with grade < 3 toxicity following the cetuximab challenge dose received a single treatment of RM-1929 {640 mg/m^2^, as determined in a previous study [34]} and a fixed light treatment dose (50 J/cm^2^ for superficial illumination and 100 J/cm fiber diffuser length for interstitial illumination). Illumination of the tumor was administered 24 ± 4 h after completion of RM-1929 infusion to allow for drug distribution within the tumor.

Red light was applied to the tumor using a frontal diffuser for surface light treatment of superficial tumors (< 1 cm thick) or a cylindrical light diffuser for interstitial placement of the diffuser into the tumors (≥ 1 cm deep). For cylindrical diffusers, 17-gauge needle catheters were placed uniformly into the tumor 1.8 ± 0.2 cm apart covering the entire tumor volume, guided by imaging methods such as ultrasound. Light was applied at an irradiation of 150 mW/cm^2^ for surface illumination and fluence rate of 400 mW/cm diffuser length for interstitial illumination, i.e. rates that do not cause thermal damage. The illumination time for frontal and cylindrical diffusers was approximately 5 min for each treated region.

Laser light treatment was given with general anesthesia based on the nature and location of tumor(s). Class IV laser precaution was required for delivery of illumination. Following RM-1929 infusion, patients were advised to avoid exposure of skin and eyes to direct sunlight or bright indoor light for at least 4 weeks, or until photosensitivity detected by minimal erythema dose (MED) evaluation had resolved.

### Study assessments

Safety was assessed at regularly scheduled time points using standard assessments. AE severity was graded according to National Cancer Institute-Common Terminology Criteria for Adverse Events (NCI-CTCAE) version 4.0.3; see Online Resource 1. DLT assessments were conducted from Day 1 to Day 7. The second and third patients were to be enrolled after DLT assessment of the first patient was completed. If a DLT occurred in one patient, an additional three patients would be added. However, if a DLT was not reported in any of the three patients, additional patients were not added. If a DLT occurred in ≤ 1 out of 6 patients, RM-1929 photoimmunotherapy was to be considered tolerable.

Skin photosensitivity was assessed by the MED on Days 1, 2, and 7, and assessments on Day 14 and at Week 4 were conducted if skin photosensitivity was noted on Day 7. Two separate 12 × 12 mm^2^ spots on the patient’s arm (in areas not used for RM-1929 administration) were exposed to light at 45 J/cm^2^ for 10 min using a solar simulator. 1 h after light illumination, erythema, eschar, and edema were measured on a 5-point scale from 0 (no finding) to 4 (severe finding).

Tumor response evaluation was conducted by central imaging review and investigators; only central imaging results are reported here. Target lesions were defined as lesions identified by the investigator that were illuminated. Non-target lesions did not undergo illumination. Baseline imaging was performed within 4 weeks prior to day 1 of treatment. An objective response was defined as a complete response or partial response (PR), the objective response rate (ORR) is the proportion of patients with complete response or partial response in the study at Week 4 follow-up tumor assessments (4 weeks ± 2 days). CT and MRI scans performed during the study were transferred to central imaging review and analyzed utilizing mRECIST (version 1.1; see Online Resource 2).

PK serum samples were collected pre-infusion, 5 min post-infusion, and at 1, 2, 4, and 24 h post-infusion. For light treatment, PK serum samples were collected at 1 and 4 h post-light exposure, and on Days 5, 7 and 14. Samples were analyzed using a validated high-performance liquid chromatography (HPLC)/fluorescence assay (Celerion, Lincoln, NE, USA) to measure concentrations of fluorescent RM-1929. Concentration values versus time data were used to estimate PK parameters using noncompartmental analysis with an intravenous infusion administration model (Phoenix^™^ WinNonlin^®^ Version 8.0).

Immunogenicity samples from all available patients were assessed for anti-drug antibodies (ADAs) on Day 1 before cetuximab challenge dose and at the Week 4 study visit. Analysis of samples for ADAs was done using a validated bridging enzyme-linked immunosorbent assay method (method number CA17849-01, Celerion, Lincoln, NE, USA). Positive ADA results in the screening assay triggered additional testing in titer and confirmatory assays.

### Statistical methods

All analyses were planned before database lock on November 6, 2018. The analysis population comprised all enrolled patients who received an infusion of RM-1929 on Day 1. Continuous endpoints were descriptively summarized, and frequencies and proportions were presented for categorical endpoints.

## Results

### Patients

Three Japanese patients were enrolled into the current study between March 26, 2018, and July 12, 2018. Given that no DLTs were observed among the first three patients that received study treatment during the DLT observation period between days 1 and 7, no additional patients were enrolled. Patient demographics and disease characteristics are shown in Table 1. All three patients were female and had good ECOG PS. All patients had failed to respond to ≥ 3 lines of prior therapy including radiation, chemotherapy, cetuximab and immunotherapy.

**Table 1:** Baseline demographics and disease characteristics for patients (*n* = 3)

Characteristic	No. of patients (%)
Age	
< 65 years	1 (33.3)
≥ 65 years	2 (66.7)
Sex	
Female	3 (100.0)
Male	0
Race	
Japanese	3 (100.0)
ECOG performance status	
0	2 (66.7)
1	1 (33.3)
2	0
Primary tumor location	
Gums^a^	1 (33.3)
External auditory canal^b^	1 (33.3)
Oropharynx^c^	1 (33.3)
Prior lines of therapy	
1	0
2	0
3	1 (33.3)
≥ 4	2 (66.7)
Prior therapy^d^	
Cancer-related surgery	1 (33.3)
Radiotherapy	3 (100.0)
Chemotherapy (platinum-based)	3 (100.0)
Immuno-/hormonal/biologic/other therapy	3 (100.0)
Cetuximab	3 (100.0)
Nivolumab	3 (100.0)

*ECOG* eastern cooperative oncology group

^a^Recurrent tumor location: soft tissue in mental/submental region

^b^Recurrent tumor location: external auditory canal

^c^Recurrent tumor location: right superficial cervical node and oropharynx

^d^Categories of prior therapy were determined by a sponsor clinical expert and were based on prior treatments reported on study

**Fig. 2:**
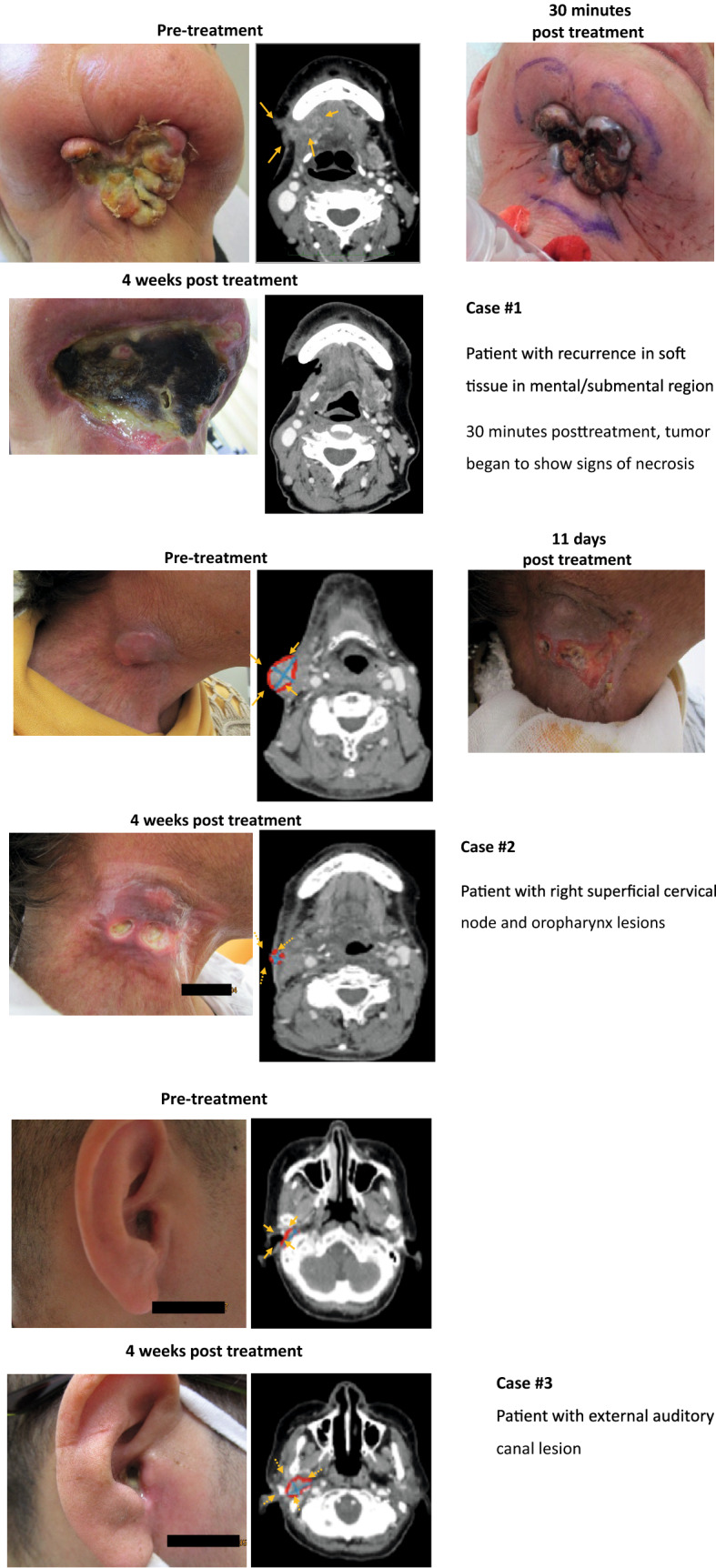
Examples of patients who received RM-1929 photoimmunotherapy for recurrent HNSCC

The recurrent tumor locations identified as target lesions were submental lesion (Fig. 2, case #1), right superficial cervical node lesion and oropharynx lesion (Fig. 2, case #2), and external auditory canal lesion (Fig. 2, case #3). One patient (case #2) had left level 2B and right level 2A lymph node metastases and multiple lung metastases that were not treated with photoimmunotherapy. A non-target nasopharyngeal lesion was also clinically noted.

### Safety

All patients experienced at least one TEAE (Table 2). None of the TEAEs were considered to be dose-limiting. The most frequently reported TEAE was application-site pain, which was seen in all three patients but resolved within the 4 week study period. Localized edema was also common, with each patient reporting either application-site edema (*n* = 1), face edema (*n* = 1), or localized edema (*n* = 1), although these events were mild in severity and resolved within 1 week of onset. All other TEAEs observed in the study were reported by only one of the three patients.

**Table 2:** TEAEs occurring in study patients (*n* = 3)

Preferred term, *n* (%)^a^	Grade 1	Grade 2	Grade 3	Grade 4
Application-site pain	2 (66.7)	0	1 (33.3)	–^b^
Application-site edema	1 (33.3)	0	0	0
Facial edema	1 (33.3)	0	0	0
Localized edema	1 (33.3)	0	0	0
Blood pressure increased	0	1 (33.3)	0	0
Gamma-glutamyl transferase increased	0	1 (33.3)	0	0
White blood cell count decreased	0	1 (33.3)	0	0
Anemia	0	1 (33.3)	0	0
Glossitis	0	1 (33.3)	0	0
Hepatic function abnormal	1 (33.3)	0	0	0
Rash generalized	0	1 (33.3)	0	0

Adverse events were coded according to the latest version of the medical dictionary for regulatory activities (MedDRA version 21.0). Adverse event severity was graded according to National Cancer Institute-Common Terminology Criteria for Adverse Events (NCI-CTCAE; version 4.0.3)

*TEAE* treatment-emergent adverse event

^a^A patient was counted only once within any TEAE by preferred term using the maximum severity grade

^b^There is no definition for grade 4 pain in the NCI-CTCAE guidelines

Of the 17 total TEAEs reported, 13 were determined by the investigator to be possibly or probably related to study treatment. Each of the three patient reports of application-site pain and localized edema were deemed probably related to study treatment. Other TEAEs that were considered possibly or probably related to study treatment were glossitis, abnormal hepatic function, increased blood pressure, and gamma-glutamyl transferase.

The majority of reported TEAEs were mild to moderate in severity with the exception of 1 report of grade 3 application-site pain, which was transient, resolved without sequelae within 24 h, and did not affect study treatment administration.

There were no DLTs, serious TEAEs, deaths, or TEAEs leading to interruption, withdrawal, or discontinuation of study treatment. Skin photosensitivity MED evaluations showed that only one of the three patients experienced very slight erythema on the Day 2 visit. No other skin photosensitivity was reported during the study. Most clinical laboratory results, vital signs, and electrocardiogram results remained within normal parameters throughout the study.

### Efficacy

Objective response based on mRECIST (version 1.1) by central review was observed in two patients, both of whom achieved a PR (Table 3). The third patient had disease progression after RM-1929 photoimmunotherapy. The clinical course of each patient is shown in Fig. 2.

**Table 3:** Overall response in patients (*n* = 3) to RM-1929 photoimmunotherapy: central review assessment

Central review assessment	No. of patients
Objective response	2
Complete response	0
Partial response	2
Stable disease	0
Disease control^a^	2
Disease progression	1

^a^Defined as patients with complete response, partial response, or stable disease

### PK and immunogenicity

Systemic exposure to RM-1929 was at maximum concentration immediately following the end of infusion and was quantifiable for approximately 14 days after dosing. RM-1929 concentrations and PK parameters were similar among the three patients (Table 4).

**Table 4:** Pharmacokinetic results (*n* = 3)

Statistical parameter	Summary pharmacokinetic parameters for RM-1929					
	*T*_1/2_ (h)	*T*_max_^a^ (h)	*C*_max_ (μg/mL)	AUC_0–∞_^b^ (h·μg/mL)	CL^b^ (mL/h/m^2^)	*V*_ss_^b^ (mL/m^2^)
Mean	60.5	2.22	370	14700	43.9	3070
SD	9.94	2.17, 2.28	17.2	1790	5.26	223
CV%	16.4	n/a	4.7	12.2	12.0	7.3

*AUC*_*0–∞*_ area under the plasma concentration–time curve, *CL* clearance, *C*_*max*_ maximum plasma concentration, *n/a* not available, *CV%* % coefficient of variation, *T*_*1/2*_ terminal half-life, *T*_*max*_ time to achieve *C*_max_, *SD* standard deviation, *V*_*ss*_ volume of distribution at steady state

^a^Median and range (minimum, maximum) relative to start of infusion

^b^Using predicted AUC_0–∞_, CL, and *V*_ss_

Serum anti-drug antibodies (ADAs) were measured to demonstrate the degree to which patients mounted an immune response against RM-1929. Immunogenicity testing showed none of the three patients tested positive for ADAs.

## Discussion

This was the first study to evaluate the safety, preliminary antitumor effects, PK, and immunogenicity of RM-1929 photoimmunotherapy in three heavily pre-treated Japanese patients with rHNSCC. Overall, RM-1929 photoimmunotherapy was well tolerated in Japanese patients. Given the mechanism of action of RM-1929 photoimmunotherapy, i.e. rapid induction of tumor necrosis, in addition to cylindrical diffusers inserted via needle catheters into deep target lesions, most treatment-related TEAEs were localized to the treatment site with application-site pain being the most frequent TEAE experienced by all three patients. Furthermore, localized edema was commonly experienced by patients, with each patient reporting either application-site edema, face edema, or localized edema. The majority of reported TEAEs were mild to moderate in severity. Although patient numbers were limited, study results suggest that the safety of RM-1929 photoimmunotherapy may be favorable compared with systemic agents used in the treatment of rHNSCC.

The majority of clinical laboratory results, vital signs, and electrocardiogram results remained within normal parameters throughout the study. Normal tissue toxicity evaluations of the tissue around the tumor that was illuminated showed that one patient experienced mild erythema and/or mild edema. In addition, one patient had very slight erythema in response to photosensitivity (MED) testing. All three patients completed study treatment without any interruptions to RM-1929 administration or light treatment.

RM-1929 photoimmunotherapy in Japanese patients showed substantial antitumor responses; two of the three patients achieved a PR (per central review assessment), which is similar to that seen in the previously presented phase I/IIa study of US patients with rHNSCC by Cognetti et al. [34]. As the study included only three patients and patients were treated with one cycle of RM-1929 photoimmunotherapy, no conclusive statement can be made with regard to the preliminary efficacy or clinical benefit of treatment. Interestingly, one patient with a PR as their best response had a recurrence at the margin of treatment, suggesting that multiple cycles of RM-1929 photoimmunotherapy could be beneficial.

Patients with advanced locoregional recurrence of head and neck cancer have poor HRQoL due to the physical effects of tumors on vital functions relating to speech and swallowing, and also detrimental esthetic effects that can have profound emotional and social impact [11]. One patient included in this study (Fig. 2, case #2) had a locoregional recurrence at the oropharynx and right cervical lymph node, which was associated with pain, and the enlarged lesion in the neck region also had a major impact on the patient’s appearance, both of which are known to affect a patient’s HRQoL. Although reduction of the oropharyngeal lesion was not confirmed, the cervical lymph node lesion decreased in size and pain was reduced by treatment. Reduction of the enlarged lesion in the neck region is likely to have had a positive impact on the patient's HRQoL, even though this was not formally evaluated in the study.

It is important to note that patients with lesions extending into bone were not excluded from this study. One patient had a local recurrence in the external auditory canal and, although the lesion inside the external auditory canal temporarily reduced in size, it increased in the lateral direction, including the surrounding bone tissue. It is presumed that the light from the diffuser could not reach the tumor because it had infiltrated the bone tissue, which may be an issue to investigate further and potentially address in the future.

PK assessment showed that systemic exposure to RM-1929 was at maximum concentration immediately after the end of infusion and was quantifiable for approximately 14 days after dosing. RM-1929 PK parameters were similar among the three treated patients. When we compared the PK profile of RM-1929 and previously reported PK findings for cetuximab [35], we found no remarkable differences in area under the plasma concentration–time curve from time zero to infinity between the two drugs, indicating that saturation of EGFR receptors may be comparable. Furthermore, the PK profiles were broadly similar between Japanese patients and the US patients who participated in the previous study of RM-1929 photoimmunotherapy [36].

Photoimmunotherapy is a novel treatment that utilizes antibodies as a targeting agent conjugated with a light-activatable dye. The requirement of the antibody to be bound to the antigen, followed by localized light activation to induce rapid cell necrosis, enables selectivity for killing antigen-expressing tumor cells. A unique feature of photoimmunotherapy is the ability to uncouple the therapeutic efficacy from that of the targeting antibody, as it does not require the cellular signaling pathway to be intact to induce necrotic cell death. Despite all three patients having previously received cetuximab, RM-1929 photoimmunotherapy was effective. Importantly, this approach could be used to treat cancers regardless of genotype/phenotype, as well as tumors refractory to other available treatments due to acquired resistance mechanisms.

In conclusion, RM-1929 photoimmunotherapy was well tolerated in three Japanese patients with rHNSCC. The safety profile and PK of RM-1929 were similar to that observed in the previously presented phase I/IIa study, and no ethnic variations were observed. Preliminary efficacy data have shown promising activity [34], and further investigation of this novel therapeutic strategy is being conducted in an ongoing global phase 3 clinical trial (NCT03769506).

## Supplementary Information

Below is the link to the electronic supplementary material.

Supplementary file1 (DOCX 17 KB)
