# Supplementary material for: A phase I, single-center, open-label study of RM-1929 photoimmunotherapy in Japanese patients with recurrent head and neck squamous cell carcinoma
Source: Int J Clin Oncol. 2021 Jun 24;26(10):1812–21. doi: 10.1007/s10147-021-01960-6 (PMC8449763; doi:10.1007/s10147-021-01960-6)
Supplement: Supplementary file 1 — Supplementary file1 (DOCX 17 KB) [file 10147_2021_1960_MOESM1_ESM.docx]

**Online resource 1**

Further details on Methodology and Results that could not be included in the main manuscript due to space restraints are provided below:

**Methods**

*Key inclusion criteria*

Key inclusion criteria were as follows: histologically confirmed rHNSCC which, in the treating physician’s opinion, could not be satisfactorily treated with surgery, radiotherapy, or platinum chemotherapy and had no other options for standard of care treatment; prior systemic platinum-based chemotherapy for rHNSCC, unless contraindicated or not recommended; life expectancy >4 months; male or female aged ≥18 years; Eastern Cooperative Oncology Group (ECOG) performance status (PS) of 0–2. Other key inclusion criteria were as follows: female patients who were not pregnant or breast feeding had to be using a medically acceptable method of birth control, be sterile, or post-menopausal; male patients had to use a medically acceptable double-barrier protection form of birth control; written informed consent from patient and/or his/her legal guardian.

*Key exclusion criteria*

Key exclusion criteria were as follows: a history of significant cetuximab infusion reactions (≥Grade 3); currently receiving chemotherapy or cetuximab therapy or radiation therapy within 4 weeks of enrollment; tumor invading a major blood vessel (such as the carotid artery) unless the vessel has been embolized, stented or surgically ligated to prevent hemorrhage; tumor not clearly shown on a computed tomography (CT) scan with contrast (magnetic resonance imaging [MRI] with gadolinium if CT not appropriate) or clinically measurable; location and extension of the tumor precludes effective photoimmunotherapy; impaired hepatic function, ALP (hepatic), AST and/or ALT >3 times ULN, and total serum bilirubin >2 mg/dL; impaired renal function (serum creatinine >2 mg/dL); uncontrolled intercurrent illness including, but not limited to, ongoing or active infection, symptomatic congestive heart failure, unstable angina pectoris, cardiac arrhythmia, or psychiatric illness/social situations that would limit compliance with study requirements. *Study assessments*

Safety was assessed at regularly scheduled timepoints using standard assessments, such as treatment-emergent AE (TEAE) monitoring, clinical laboratory tests, vital signs, electrocardiograms, physical exams, and concomitant medications monitoring. AEs were coded according to the Medical Dictionary for Regulatory Activities (version 21.0).

**Results**

*Patients*

Three Japanese patients were enrolled into the current study between March 26, 2018, and July 12, 2018. All three patients were female, aged in their 50s, 60s, or 70s, and had good ECOG PS (ECOG PS: 0 in two patients, and one in one patient).

**Online resource 2**

**Modified Response Evaluation Criteria in Solid Tumors (mRECIST) 1.1 assessment criteria**

The RECIST 1.1. criteria of 2009 standardize image interpretation for solid tumor indications. RECIST 1.1 bases the assessments of response and progression upon unidimensional measurements of target lesions, qualitative assessments of other tumor burden categorized as nontarget lesions, and the appearance of any new lesions. The implementation of modified RECIST 1.1 for the central reads of the RM-1929-102 study is described within this appendix and includes the following adaptions/modification to the published criteria:

- The therapy administered for the RM-1929-102 protocol is localized to those tumors that receive targeted light treatment. Due to the localized nature of this treatment, RECIST 1.1 was modified as follows: The central review will select treated tumors as mRECIST 1.1 target lesions. The information about treated tumors and annotated images, if available, will be provided to central review as clinical data to allow for identification of the treated tumors. The treated tumors were identified as disease by the investigator site based upon pathology and will therefore be selected as target lesions regardless of their size or the quantity of target lesions. This means that the RECIST 1.1 criterion for a maximum of 5 target lesions with a maximum of 2 per organ does not apply, nor does the criterion for minimum target lesion size (≥10 mm in longest diameter for non-nodal lesions or ≥15 mm in short axis for nodes).
- mRECIST 1.1 target lesions will be measured by volume (if possible) and the unidimensional mRECIST 1.1 measurement (longest diameter for non-nodal lesions and short axis for nodes) will be derived using this volume. If volume cannot be generated, the mRECIST 1.1 diameter measurements will be generated by the reviewer him/herself. The volume will be reported for purposes of post-review analysis but will not contribute to the Sum of Diameters (SOD) or overall response assessments. Considering that the volume does not impact the overall response assessments, this is not a modification to the criteria but rather an adaptation that will allow additional lesion data to be captured during the review.
